# Leaf Eco-Physiological Profile and Berries Technological Traits on Potted *Vitis vinifera* L. cv Pinot Noir Subordinated to Zeolite Treatments under Drought Stress

**DOI:** 10.3390/plants11131735

**Published:** 2022-06-29

**Authors:** Eleonora Cataldo, Maddalena Fucile, Giovan Battista Mattii

**Affiliations:** Department of Agriculture, Food, Environment and Forestry (DAGRI), University of Florence, 50019 Sesto Fiorentino, FI, Italy; maddalena.fucile@unifi.it (M.F.); giovanbattista.mattii@unifi.it (G.B.M.)

**Keywords:** global warming, leaf temperature, low water availability, clinoptilolite, grape quality, photosynthesis, water potential

## Abstract

In Mediterranean areas, extreme weather conditions such as high diurnal temperatures during the growing season could tweak vine physiology and metabolism, affecting grapes’ quality. Moreover, uncertainty in spatial and temporal distribution precipitation is an issue for the water resources of the vineyards, forcing the winemakers to continuously face an increasing water demand in recent decades, which has led them to non-sustainable choices for ambient (i.e., irrigation solutions). The aspiration of this experiment was to explore the effects of zeolite treatments (clinoptilolite type) on *Vitis vinifera* L. (potted vines) ecophysiology and berry metabolism under two water regimes. The plants were subordinated to two different predawn water potential regimes (0 ≤ ΨPD ≤ −0.4, WWCtrl and −0.4 ≤ ΨPD ≤ −0.9, WSCtrl), both associated with zeolite treatments (WWt and WSt). Gas exchanges, predawn and midday stem water potential, chlorophyll fluorescence, temperature, and relative water content were overseen on leaves at veraison, maturation, and harvest. Technological analyses were performed on the berries. Moreover, data were analyzed with principal component analysis and Pearson’s correlations. This experiment supplies new evidence that zeolite applications could impact both physiological profiles (higher photosynthesis and stomatal conductance) as well as berry skin metabolism (sugar and size) of vines, giving a better skill to counteract low water availability during the season and maintaining a better hydraulic conductivity.

## 1. Introduction

The concentration of greenhouse gases (GHGs) in the atmosphere (such as carbon dioxide (CO_2_), nitrous oxide (N_2_O), and methane (CH_4_)) has rapidly increased in the last century mainly due to the continuous and copious reversal in the atmosphere of combustion products provided by energy systems that employ, especially, fossil fuels (methane, coal, and oil) [1]. Agricultural ecosystems are important sinks and sources of GHGs by producing and consuming them in biological processes (such as nitrification, denitrification, net photosynthesis, respiration, decomposition, methanogenesis, and CH_4_ oxidation) [2]. A direct consequence of this phenomenon is the growth of the planet’s average temperature [3].

The Intergovernmental Panel on Climate Change (IPCC) is currently in its Sixth Assessment Report (AR6). The results of the research group show that in the coming decades an increase in climate change is expected in all regions of the world in multiple ways [4]. The report’s analysis unveils that at 1.5 °C of global warming, it is forecasting a swell in the number of heatwaves, extended hot seasons, and fleeting chilly seasons. While at 2.0 °C global warming, the warm–extreme temperatures would climb to critical tolerance thresholds for agriculture more often [5]. However, the temperatures are not the only component at play. Climate change is driving many other alterations in different regions, and all will intensify with further warming. These embody modifications in annual rainfall rates, winds, snow, coastal districts, and oceans level [6].

History showed that wine grape growing regions thrived when the climate was most conductive in anthropic activity and that the unpredictable shifts occurred owing to climate vicissitudes, making production and quality more taxing [7,8,9,10]. In viticulture, climate represents a terroir component having a deep effect on the capacity of a region or territory to trigger excellent berries and consequently wine [11,12]. Sundry studies identify changes in grapevine growth characteristics and grapes quality as an effect of climate warming and of the frequency/intensity increase in extreme meteorological events (droughts, hail, high temperatures, tropical nights, and flooding) [13,14,15,16,17,18].

Indeed, higher diurnal temperatures sway the plant’s ecophysiology and phenology by shifting the phenological stages to earlier times, including the ripening phase shifting to warmer periods that affect the grape composition, by enhancing sugar content, diminishing total acidity, and unbalancing the aroma compounds [19,20,21].

Moreover, uncertainty in spatial and temporal precipitation distribution is a notable issue for the water resources of the vineyards, forcing the winemakers to continuously face an increasing water demand in recent decades, which has led to an increase in the gap between supply and demand for water. Strengthening this split inevitably led to a serious consideration of the basics of water resource planning by adopting appropriate management strategies such as emergency irrigation [22,23].

It was shown that the water deficit induced lipid peroxidation, chlorophyll bleaching, and molecular antioxidants loss (i.e., ascorbate, glutathione, α-tocopherol, and carotenoids) by decreasing the activities of active oxygen-processing enzymes, such as ascorbate peroxidase (APOX), superoxide dismutase (SOD), catalase (CAT), and nonspecific peroxidase (POX) [24,25,26,27,28]. In addition to the stomatal limitation of photosynthesis under water stress, non-stomatal effects occur under severe stress [29,30,31]; extreme environmental conditions, above 43 °C temperatures, can damage the photosynthetic machinery (photosystem II (PSII)) reversibly or irreversibly [32,33]. The plant’s responses under water deficit stress and high temperatures occur from the leaf level to the whole-vine level, including carbon assimilation and the allocation of photoassimilates (e.g., leaf proline accumulation, for reducing cell acidity and the release of ammonia toxicity, the presence of several reactive oxygen species as mediators of signal transduction, a high hydrogen peroxide content, and 3-hexenal and (E)-2-hexenal) [34,35].

Although putting concrete numbers on this affirmation is extremely difficult, many studies argue that irrigation is not a sustainable and resilient solution, with a view to expanding the areas planted with vineyards [36,37].

The resilience-broad concept, defined among authors in several different ways, forks out a sovereign framework for examining critical ecosystem transitions in response to environmental change [38,39]. Here, we focus on ecological resilience, pioneered by Holling (1973) [40] and defined as the skill of “a system to experience shocks while retaining essentially the same structure, function, feedbacks, and therefore identity” [41]. In this definition, resilience is calculated as the perturbation amount (such as climate variables metamorphosis) that a system can absorb before reaching a tipping pinnacle or margin beyond which it transitions into an alternative condition causing an undesirable locked state (e.g., hysteretic system) [42,43].

The logical solution is a detailed mechanistic and physiologic understanding of the problem in order to better design and target vineyard management approaches [44] for the development of new solutions against drought with the aim of corroborating the vineyard ecosystem and increasing its resilience over time.

This increasing demand for vineyard protection against environmental adversity requires an increase in the production of materials to be used in natural-based viticulture such as zeolites [45]. As non-toxic, ecologically worthwhile, and bearable materials, the natural zeolites, owing to their characteristics (structural, sorption, and ion exchange properties) are well suited for agricultural employees, both animal and plant production [46]. 

Zeolites (from the Greek words, ζέω, “boil” and λίθος, “stone” [47]) are alumino-silicate minerals that have a molecular sieve action owing to their framework (open channel network) [48]; these minerals are constituted by TO4 tetrahedra linked with oxygen atoms sharing the negative charge created by the presence of AlO^2-^ and SiO^2-^ balanced by cations (i.e., Na^+^, K^+^, Rb^+^, Cs^+^, Mg^2+^, and Ca^2+^) that neutralize the charge deficiency [49]. Due to their high adsorption capacities in the dehydrated state and the high ion-exchange capacity, zeolites can help in the absorption and retention of plant nutrients, herbicides, supplemented micronutrients, fungicides, fertilizer, pesticides, and water [50,51]. Owing to porous and capillary suction properties, zeolite forwards soil water retention and infiltration and operates as a natural wetting element [52,53].

For these reasons, the proposals of this project were to test if zeolite treatments were able to positively sway grapevine ecophysiology by promoting a higher tolerance to drought in controlled conditions, and to investigate the effect on technological berry parameters, analyzing grape development.

In order to achieve these goals, the comparison between grapevines treated with zeolite (clinoptilolite type) and non-treated ones was set up on Pinot noir cv. potted grapevines (*Vitis vinifera* L.), both subordinated to two different irrigation regimes.

## 2. Results

### 2.1. Meteorological Parameters 

**Figure 1:**
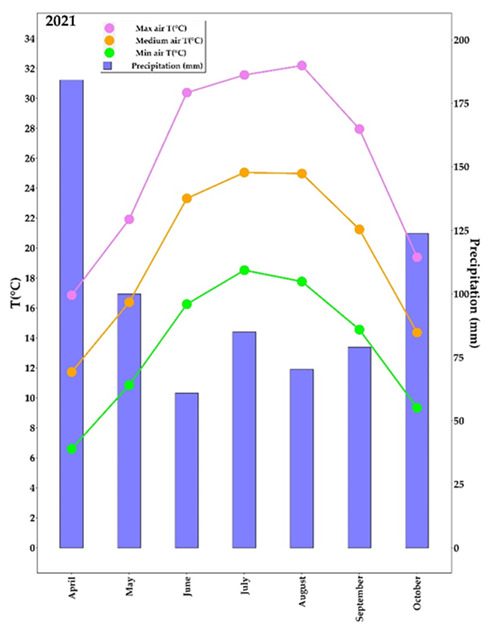
Meteorological parameters of the experiment location. Monthly averages of mean, maximum, and minimum air temperature (°C) and precipitation (mm) were measured from April to October.

The total average means, maximum, and minimum air temperatures, recorded from bud break to leaf fall (April–October), were 19.58 °C, 25.75 °C, and 13.41 °C, respectively (Figure 1).

The two hottest months of this period were July and August (maximum temperatures were always above 35 °C in the last seven days of July and in the middle eleven days of August). The hottest days were the 7th of July (T_max_ = 35.6 °C), the 9th of August (T_max_ = 35.8 °C), the 10th of August (T_max_ = 36.0 °C), the 11th of August (T_max_ = 36.8 °C), and the 12th of August (T_max_ = 39.3 °C) when maximum and minimum air temperatures were higher than the corresponding monthly averages. 

### 2.2. Leaf Gas Exchange, Chlorophyll Fluorescence, Water Potential (Leaf, Stem, and Pre-Dawn), Relative Water Content, Plant Hydraulic Conductance, Intercepted Photosynthetically Active Radiation, and Leaf Temperature

**Figure 2:**
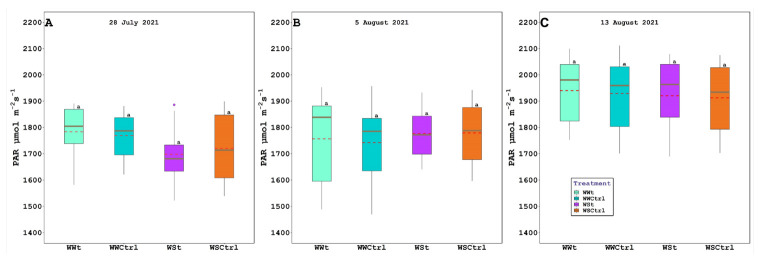
Photosynthetically active radiation (PAR; µmol m*^−^*^2^s*^−^*^1^) in *Vitis vinifera* (Pinot Noir cv.) treated with Zeolite (t) and untreated plants (Ctrl), under two irrigation regimes (WW, well-watered; WS, water-stressed); (**A**). 28 July 2021, (**B**). 5 August 2021, and (**C**). 13 August 2021. The box bounds show the 25 and 75 percentiles, and the error bars the 90 and 10 percentiles. The black continuous and red discontinuous lines inside the boxes represent medians and means, respectively. Outliers are represented as color dots. Average values with the same letter in each figure indicate no significant differences between plots (*p* < 0.05).

At the level of photosynthetically active radiation (PAR) during all measurements, no significant difference was found (Figure 2).

**Figure 3:**
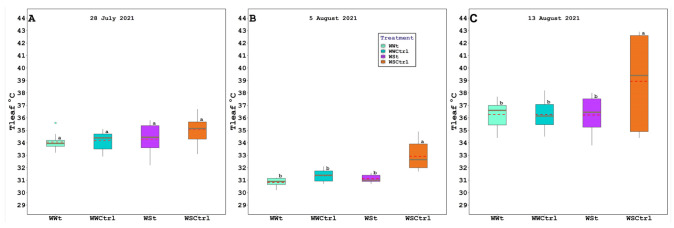
Leaf temperature (Tleaf; °C) in *Vitis vinifera* (Pinot Noir cv.) treated with Zeolite (t) and untreated plants (Ctrl), under two irrigation regimes (WW, well-watered; WS, water-stressed); (**A**). 28 July 2021, (**B**). 5 August 2021, and (**C**). 13 August 2021. The box bounds show the 25 and 75 percentiles, and the error bars the 90 and 10 percentiles. The black continuous and red discontinuous lines inside the boxes represent medians and means, respectively. Outliers are represented as color dots. Average values with the same letter in each figure indicate no significant differences between plots (*p* < 0.05).

**Figure 4:**
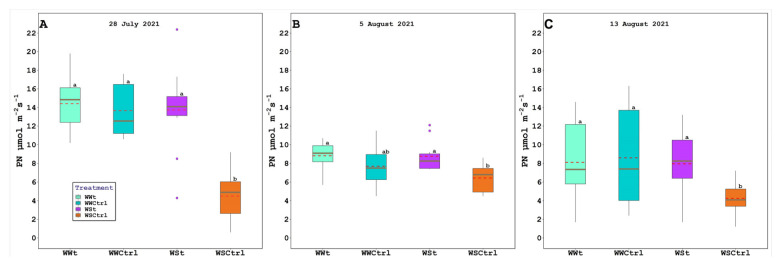
Net photosynthesis (PN; µmol m*^−^*^2^s*^−^*^1^) in *Vitis vinifera* (Pinot Noir cv.) treated with Zeolite (t) and untreated plants (Ctrl), under two irrigation regimes (WW, well-watered; WS, water-stressed); (**A**). 28 July 2021, (**B**). 5 August 2021, and (**C**). 13 August 2021. The box bounds show the 25 and 75 percentiles, and the error bars the 90 and 10 percentiles. The black continuous and red discontinuous lines inside the boxes represent medians and means, respectively. Outliers are represented as color dots. Average values with the same letter in each figure indicate no significant differences between plots (*p* < 0.05).

**Figure 5:**
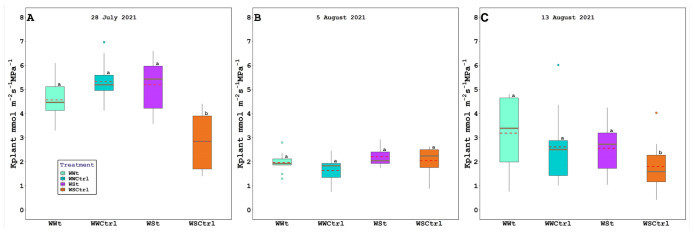
Plant hydraulic conductivity (Kplant; mmol m*^−^*^2^s*^−^*^1^ MPa*^−^*^1^) in *Vitis vinifera* treated with Zeolite (t) and untreated plants (Ctrl), under two irrigation regimes (WW, well-watered; WS, water-stressed); (**A**). 28 July 2021, (**B**). 5 August 2021, and (**C**). 13 August 2021. The box bounds show the 25 and 75 percentiles, and the error bars the 90 and 10 percentiles. The black continuous and red discontinuous lines inside the boxes represent medians and means, respectively. Outliers are represented as color dots. Average values with the same letter in each figure indicate no significant differences between plots (*p* < 0.05).

Leaf gas exchange, chlorophyll fluorescence, water potential (leaf, stem, and pre-dawn), relative water content, and plant hydraulic conductance are presented below (Figure 3, Figure 4 and Figure 5; Table 1 and Table 2).

Lower Pn and gs values were observed in WSCtrl compared to the other treatments. In general, no difference was found between WWt, WWCtrl, and WSt for these parameters.

On August 5 and August 13, WSCtrl plants exhibited significantly higher leaf temperature values than irrigated and/or treated plants, while WUE was largely unaffected by zeolite treatments. At the same sampling time, the water-stressed vines maintained lower E levels than the other treatments.

ΨPD and Ψstem were largely influenced by zeolite treatments and irrigation. Under water stress conditions, WSt vines showed higher ΨPD and Ψstem values than WSCtrl. Furthermore, the RWC was significantly higher in WWt, WWCtrl, and WSt than in the WSCtrl plants.

*Fv/Fm* showed significant changes due to treatment only on 5 August 2021 (the WSCtrl treatment showed significantly lower values than the other treatments).

### 2.3. Grape Composition

**Figure 6:**
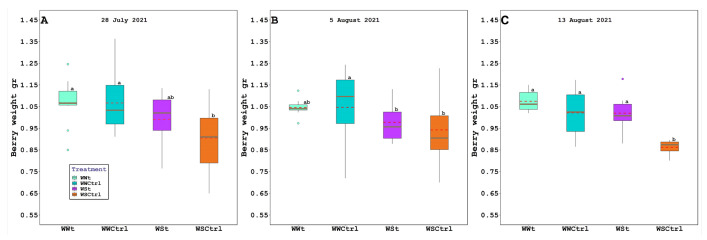
Berry weight (bw; gr) in *Vitis vinifera* (Pinot Noir cv.) treated with Zeolite (t) and untreated plants (Ctrl), under two irrigation regimes (WW, well-watered; WS, water-stressed); (**A**). 28 July 2021, (**B**). 5 August 2021, and (**C**). 13 August 2021. The box bounds show the 25 and 75 percentiles, and the error bars the 90 and 10 percentiles. The black continuous and red discontinuous lines inside the boxes represent medians and means, respectively. Outliers are represented as color dots. Average values with the same letter in each figure indicate no significant differences between plots (*p* < 0.05).

The irrigation regime and zeolite treatment induced significant differences (*p* ≤ 0.05) in berry weight, sugar content, and titratable acidity (Figure 6 and Table 3). 

At harvest, the berry weight was −19.62%, −15.68%, and −15.68% lower in WSCtrl than in WWt, WWCtrl, and WSt vines, respectively.

At harvest, the sugar content was +4.09%, +10.26%, and +0.55% higher in WSCtrl than in WWt, WWCtrl, and WSt vines, respectively.

Zeolite treatments did not induce significant effects on the pH parameter in both water regimes.

### 2.4. Pearson’s Correlation

**Figure 7:**
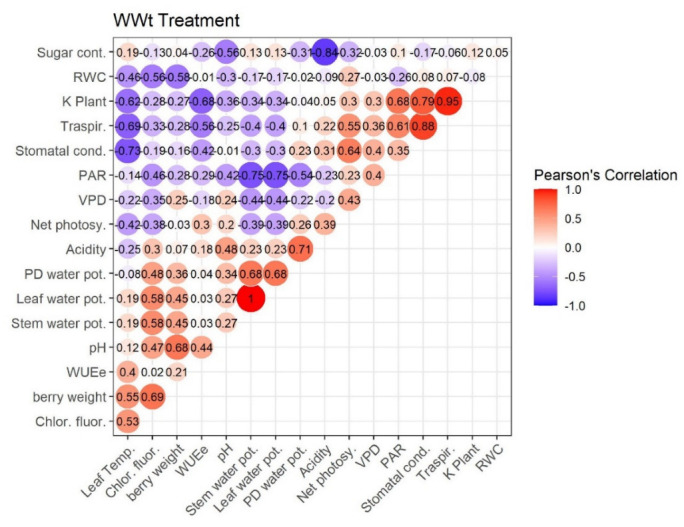
Pearson’s Correlation. WWt Treatment (Well-watered + Zeolite). Ecophysiological and technological correlations between the pairs of traits were analyzed. Correlations were calculated from the mean values of each of the four treatments characterized. Positive correlations are displayed in red and negative correlations in violet. The color intensity and the size of the circle are proportional to the correlation coefficients. The grey background boxes illustrate the significant values at the level of *p* < 0.05 (two-tailed).

**Figure 8:**
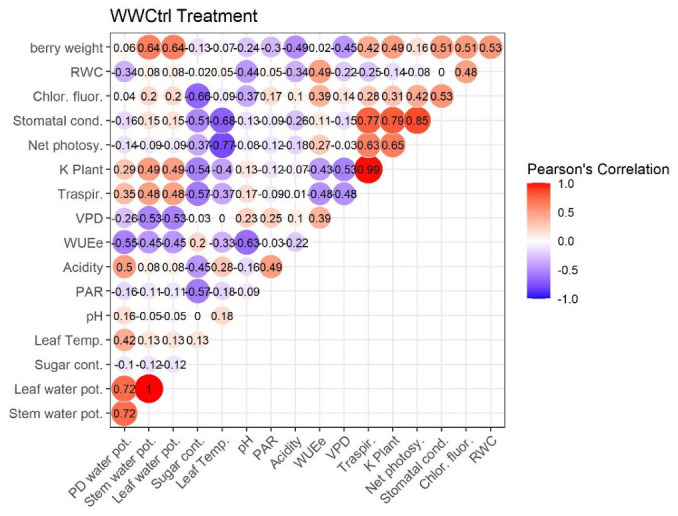
Pearson’s Correlation. WWCtrl Treatment (Well-watered). Ecophysiological and technological correlations between the pairs of traits were analyzed. Correlations were calculated from the mean values of each of the four treatments characterized. Positive correlations are displayed in red and negative correlations in violet. The color intensity and the size of the circle are proportional to the correlation coefficients. The grey background boxes illustrate the significant values at the level of *p* < 0.05 (two-tailed).

**Figure 9:**
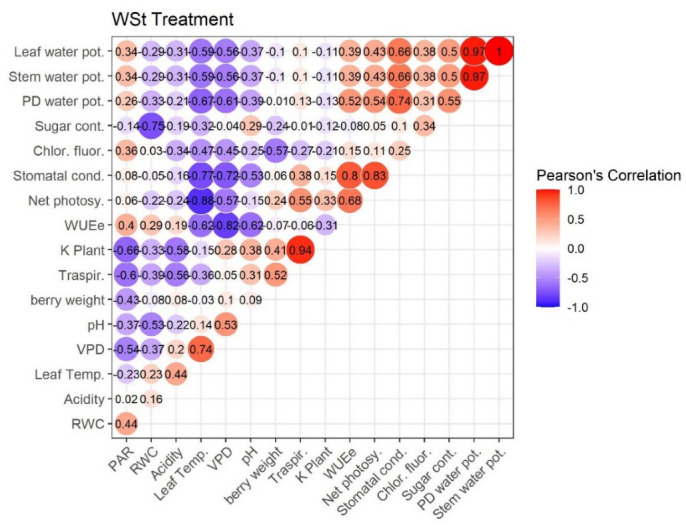
Pearson’s Correlation. WSt Treatment (Water stress + Zeolite). Ecophysiological and technological correlations between the pairs of traits were analyzed. Correlations were calculated from the mean values of each of the four treatments characterized. Positive correlations are displayed in red and negative correlations in violet. The color intensity and the size of the circle are proportional to the correlation coefficients. The grey background boxes illustrate the significant values at the level of *p* < 0.05 (two-tailed).

**Figure 10:**
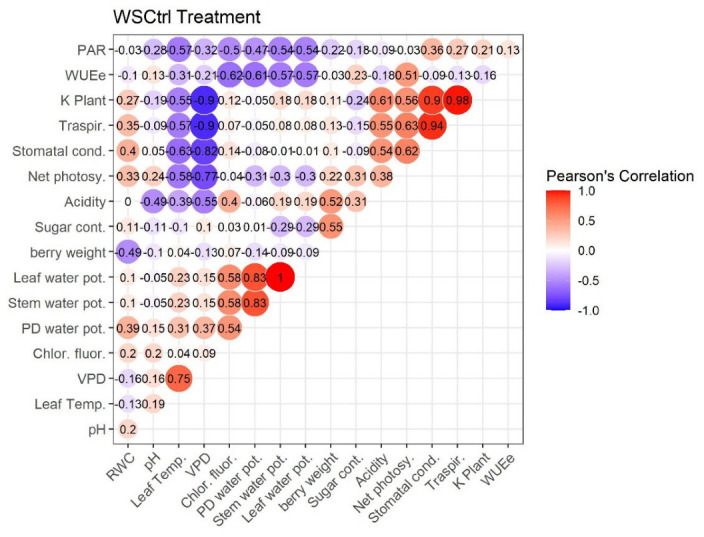
Pearson’s Correlation. WSCtrl Treatment (Water stress). Ecophysiological and technological correlations between the pairs of traits were analyzed. Correlations were calculated from the mean values of each of the four treatments characterized. Positive correlations are displayed in red and negative correlations in violet. The color intensity and the size of the circle are proportional to the correlation coefficients. The grey background boxes illustrate the significant values at the level of *p* < 0.05 (two-tailed).

Figure 7, Figure 8, Figure 9 and Figure 10 represent Pearson’s correlations for each treatment (WWt, WWCtrl, WSt, and WSCtrl).

In general, in all treatments, significant positive correlations were identified between the following character pairs: Kplant–Traspir, PD water pot–Stem water pot, PD water pot–Leaf water pot, and Traspir–gs. Moreover, a (very close) negative correlation was identified between Sugar–Acidity and Net photosy–Leaf temp.

### 2.5. Principal Component Analysis (PCA)

**Figure 11:**
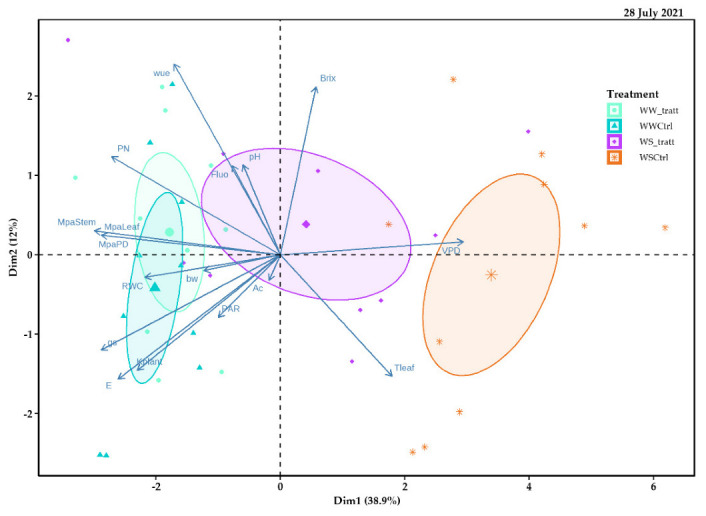
Relationship between principal component 1 (Dim1) and principal component 2 (Dim2) for response variables analyzed in field experiments with Pinot Noir cultivar subjected to different treatments (WWt, WWCtrl, WSt, and WSCtrl) during the 2021 season (28 July 2021).

Principal component (PC) 1 (Dim1) of the 28 July 2021 explained 38.9% of the data variability and allowed for visualizing different treatment behaviors (delimited by different colored ellipses) in comparison, carried out based on the response variables. The WSCtrl treatment was to the right of the spatial distribution and positively related to a production variable (i.e., sugar content (°Brix)), VPD, and Tleaf, and negatively related to ecophysiological variables such as PN, E, PAR, and stomatal conductance (gs). Instead, principal component 2 (Dim2) explained 12.0% of the data variability (Figure 11). 

**Figure 12:**
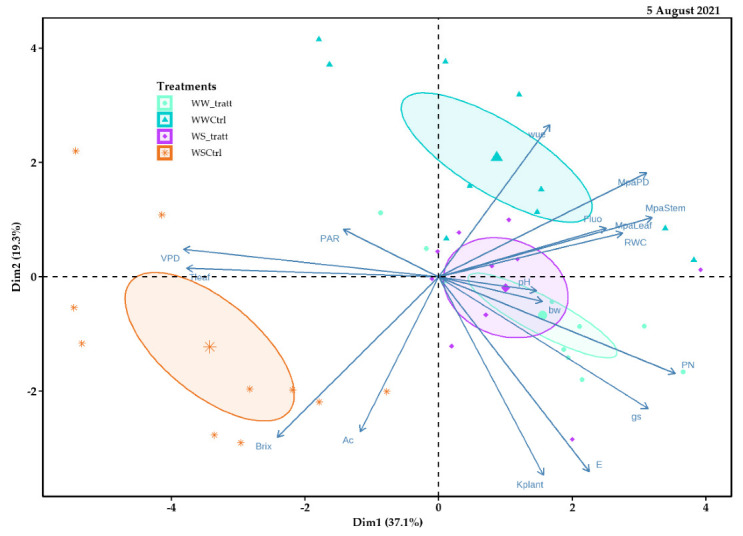
Relationship between principal component 1 (Dim1) and principal component 2 (Dim2) for response variables analyzed in field experiments with Pinot Noir cultivar subjected to different treatments (WWt, WW, WSt, and WS) during the 2021 season (5 August 2021).

Principal component 1 (Dim1) of the 5 August 2021 explained 37.1% of the data variability and allowed for visualizing different treatment behaviors (delimited by different colored ellipses) in comparison, carried out based on the response variables. The WWt and WSt treatments were similarly distributed to the right of the spatial distribution. These treatments were negatively related to production variables such as sugar content (°Brix) and acidity (Ac), and positively related to water potential parameters such as MPaPD, MPaLeaf, and MPaStem. Instead, principal component 2 (Dim2) explained 19.3% of the data variability (Figure 12).

**Figure 13:**
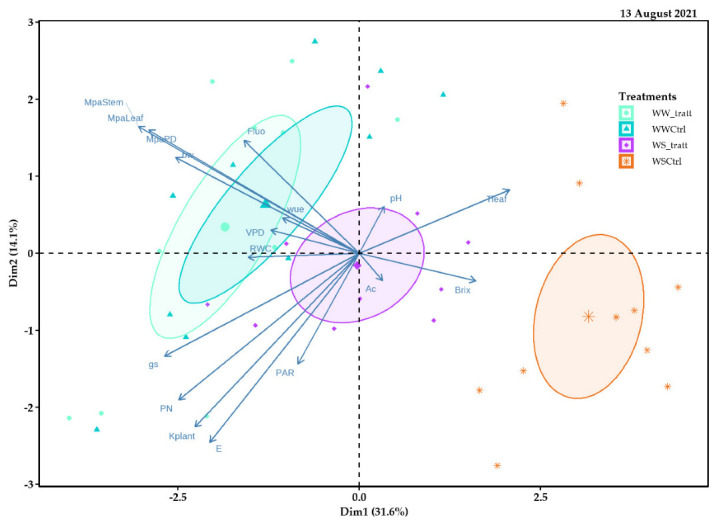
Relationship between principal component 1 (Dim1) and principal component 2 (Dim2) for response variables analyzed in field experiments with Pinot Noir cultivar subjected to different treatments (WWt, WWCtrl, WSt, and WSCtrl) during the 2021 season (13 August 2021).

Principal component 1 (Dim1) of the 13 August 2021 explained 31.6% of the data variability and allowed for visualizing different treatment behaviors (delimited by different colored ellipses) in comparison, carried out based on the response variables. The WWt and WWCtrl treatments were similarly distributed to the left of the spatial distribution; they were negatively related to ecophysiological variables such as PN, gs, E, and KPlant, and positively related to technological parameters such as °Brix, pH, and Ac. While the WSCtrl treatment was positioned to the right of the spatial distribution. Instead, principal component 2 (Dim2) explained 14.1% of the data variability (Figure 13).

## 3. Discussion

Important alterations in ecophysiological reactions are regularly noted in grapevines under different statuses of water stress, such as with a severe limitation in vine water uptake, a drop-in the leaf stomatal conductance (e.g., on 13 August 2021 WSCtrl recorded 54.70 mmol m^−2^s^−1^), and in the photosynthetic rate (e.g., on 13 August 2021 WSCtrl recorded 6.43 µmol m^−2^s^−1^) [54,55,56], which often influence berry sugar accumulation and yield [57]. Given the absence of works with applications of zeolites to the soil in the vineyard, the possible changes in ecophysiological responses have not been found in grapevines under soil zeolite treatments yet.

In our study, as expected, net photosynthesis (PN), stomatal conductance (gs), predawn water potential (ΨPD), stem water potential (Ψstem), and relative water content (RWC) were significantly weakened by drought in WSCtrl grapevines with respect to WWCtrl, WWt, and WSt ones. Regarding the net photosynthesis on a single leaf, the zeolite treatment influenced all the sampling dates in the water potential (pre-dawn and stem) differences due to the treatment observed on 5 and 13 August, while in the RWC, the differences were found on 5 August. Probably, the stressed-treated vines (WSt) improved their performance due to zeolite properties by enhancing the nutrient use efficiency, heightening the phosphorus (P) availability from phosphate rocks, and the utilization of ammonium nitrogen (NH_4_^+^–N) and nitrate nitrogen (NO_3_^—^N), reducing losses by the leaching of exchangeable cations, in particular K^+^, and functioning also as a slow-release fertilizer [49,58] (e.g., positive effects on lettuce [59] and tomato [60]). In our study, maybe by increasing the soil water holding capacity [61], zeolite application increased the water use efficiency (WUE) (i.e., on 28 July 2021 WWt = 3.21 µmol mmol^−1^, WSt = 3.00 µmol mmol^−1^, WWCtrl = 2.67 µmol mmol^−1^, and WSCtrl = 1.64 µmol mmol^−1^).

Owing to the zeolitic skill to retain water [62,63], vines treated with clinoptilolite (WWt and WSt) evidenced significantly inferior leaf temperatures (TLeaf) than WSCtrl plants. The following diminutions were registered during the season: −2.91% WWt and −2.21% WSt on July 28th; −6.81% WWt and −5.78% WSt on August 5th; and −7.33% WWt and −7.47% WSt on August 13th. Here we hypothesize that the application in the pot made the roots more insulated and humid in order to isolate the leaves from photo-oxidative damage [64] (see also the improved *Fv/Fm* ratio of irrigated and treated plants compared to stressed ones). It is hypothesized that the balancing of this parameter allowed the reduction of stress, in turn improving the stomatal conductance parameter (as emerges from gs/Tleaf Pearson’s Correlation: 0.73 WWt, 0.68 WWCtrl, 0.77 WSt, and 0.63 WSt). In addition, it is well known that transpiration serves as a heat dissipation mechanism in plants. Therefore, the significant increase in Tleaf in the WSCtrl treatment compared with the other treatments may be related to the significant reduction in leaf transpiration observed in the WSCtrl treatment (as emerges from gs/E Pearson’s Correlation: 0.88 WWt, 0.77 WWCtrl, 0.38 WSt, and 0.94 WSt).

In our study, the water (WWCtrl), the synergy of water/zeolite (WWt), and only zeolite application (WSt) positively influenced water stress. In fact, by improving the physical and chemical properties of the soil, (infiltration rate, saturated hydraulic conductivity, and water holding capacity [65]), many studies on other species showed that water deficit stress can be mitigated by soil applications of zeolite such as in *Phaseolus vulgaris* L. [66], *Salvia officinalis* L. [67], *Dracocephalum moldavica* L. [68], *Brassica napus* L. [69], and *Helianthus annuus* L. [70]. The effectiveness of zeolite treatment on vine ecophysiology could be also discerned at the first stage (28 July 2021) under water stress conditions, since treated vines maintained higher water potentials (ΨPD, Ψstem) than in WSCtrl vines and at the third stage (13 August 2021) where the high air temperature (38.5 °C) induced a strong decrease in RWC; however, this dwindle was more evident in nontreated grapevines (confirming the hypothesis of a positive effect of zeolite in preventing excessive leaf dehydration [71]). However, the effect of zeolite under water deficit conditions maintaining higher water potentials than in the WSCtrl treatment was more evident in the second and third stages (5 and 13 August).

The treatment’s effect on potted grapevine water status continued and was amplificated with water stress evolution. Indeed, on 13 August 2021, WSt vines revealed a better Kplant than WSCtrl plants. The allowance of a satisfactory Kplant could have countenanced WSt leaf to avoid stomatal closure and an excessive constraint in carbon gain [72]. Moreover, since the Kplant fall-off is correlated not only to transpiration (i.e., Kplant/E Pearson’s Correlation: 0.95 WWt, 0.99 WWCtrl, 0.94 WSt, 0.98 WSt) but also to a hydraulic dysfunction, our conclusions lead us to believe that WSt leaves are less susceptible to conduit embolism and hence collapse [73] (important in anisohydric Pinot noir cv., more unguarded to hydraulic breakdown [74]).

Stressed and untreated vines (WSCtrl) showed significantly more negative water potentials (Ψ) than WWt and WSt plants. In predawn water potential during the season, the following decreases were recorded for WS treatment, respectively: −70.32% and −14.28% on July 28th; −45.74% and −31.91% on August 5th; and −63.63% and −30.30% on August 13th.

Finally, treatments significantly affected sugar accumulation and berry weight. 

This result is not in agreement with the results observed by Salvi et al. (2020) [75] on Pinot noir cv. in pot conditions, where the authors did not find differences in sugar maybe for restricted carbohydrates reserves stored in grapevine roots. In particular, it can be seen that in the last survey at harvest (13 August 2021) the WSt treatment retains the same sugar level as WSCtrl but has a significantly higher weight of the berry (WSCtrl = 0.86 gr vs. WSt = 1.03 gr). As regards the concentration of sugar at harvest, there is a greater Brix degree in the WWt treatment (19.30 °Brix) compared to the corresponding WWCtrl (18.22 °Brix) not due to the concentration effect (same weight of the berry). Whereas, pH parameter was not affected by treatments. It is assumed that the increase in the berry weight in plants treated with zeolites is attributable to the zeolitic properties of corroborating the water performance of the plant (more hydrated berries) [62]. As regards the tendency of the increase in sugar in WWt plants compared to WWCtrl, an interaction of the zeolite in the metabolic pathways of sugar accumulation is supposed. It is therefore believed that further investigations should be made above all an investigation activity of sugars accumulated in the vacuoles, studying the activity of sucrose-metabolizing enzymes, sucrose transporters, and monosaccharide transporters.

However, there are a few precautions (experiment done in pots) to be taken for extrapolating the results to vines under field conditions such as for example, an in-depth study of the orography of the territory, multi-year pluviometric analysis, and complete soil analyses, in order to better evaluate the feasibility of applying the mineral.

## 4. Materials and Methods

### 4.1. Location, Meteorological Parameters, and Experiment Design

This experiment was carried out during the 2021 growing season on Pinot noir (Entav 115 clone) 12-year-old homogeneous potted grapevines (shoot length average after topping 95.0 cm, leaves per vine average number 105.0; ten buds per vine; average canopy leaf area/vine 1.17 m^2^) (*Vitis vinifera* L.). Vines were grafted onto 1103 Paulsen *V. berlandieri* × *V. rupestris* rootstock, trained on a vertical shoot positioned trellis, with spur cordon pruning, and grown outdoor in Arezzo, Italy (Lat. 43°27′47″ N 11°52′41″ E; 296 m a.s.l.).

Daily values of mean/minimum/maximum air temperatures (°C) and mean/minimum/maximum humidity (%) values were recorded from April to October using a nearby meteorological station (Bresser WeatherCenter 7002500, Rhede, Germany).

Pots had a holding capacity of 80 L; they were filled with clay-loam soil (clay 39%; silt 34%; and sand 27%), with a volumetric soil water content (SWC) of ∼35.0% at field capacity. During February/March, every year, each pot was fertilized with 50 g of Nitrophoska controlled-release fertilizer (15N–9P–15K) (Eurochem Agro, MB, Italy). To escape an overmuch soil over warming that can negatively impact roots, and preserve a reliable temperature, all pots were sheltered by a white painting.

From the beginning of July until harvest, 20 plants were maintained at 90% of maximum water availability (WWCtrl, well-watered grapevines), 10 of which were treated with zeolite (0 ≤ ΨPD ≤ −0.4), while the other 20 vines were subjected to a water deficit at 40% of maximum water availability (WSCtrl, water-stressed), 10 of which were treated with zeolite (−0.4 ≤ ΨPD ≤ −0.9) [75,76]. During water restriction, the plant container’s surface was curtained with aluminum foils to impede rainfall intervention and to downsize evaporation. The contributed water per pot was calculated by monitoring every day the soil moisture (volumetric content) by time-domain reflectometry (Soil Moisture Equipment Corporation, CA, USA) with 30 cm long electrodes located in the pots. Water was supplied at 2-day intervals with drip irrigation emitters.

The zeolite application (clinoptilolite 80%, granulometry 0.2–2.5 mm; Zeocel, DND Biotech srl, PI, Italy) took place on February 8, 2021, at a dose of 1.0 kg per pot [77,78].

At three different stages, on 10 vines per treatment, eco-physiological measurements and berries samplings were conducted: Time1 (full-veraison, Eichorn and Lorenz (E-L) stage 35; 28 July 2021), Time2 (maturation; E-L stage 37; 5 August 2021), and Time3 (harvest; E-L stage 38; 13 August 2021) [79].

### 4.2. Leaf Gas Exchange, Chlorophyll Fluorescence, Water Potential (Leaf, Stem, and Pre-Dawn), Relative Water Content, Plant Hydraulic Conductance, Intercepted Photosynthetically Active Radiation, and Leaf Temperature

At Time1 (full-veraison; 28 July 2021), Time2 (maturation; 5 August 2021), and Time3 (harvest; 13 August 2021), net photosynthesis (Pn), leaf temperature (LTemp), transpiration rate (E), stomatal conductance (*gs*), vapor pressure deficit (VPD), and photosynthetically active radiation (PAR) were measured on 10 fully developed and healthy leaves per treatment (one each grapevine/10 replicates) adopting Ciras 3 (ambient light and 400ppm CO_2_ [80]), a portable infrared gas analyzer (PP Systems, MA, USA). Extrinsic water use efficiency (WUEe) was calculated as follows: Pn and E ratio [81].

On the same leaves chosen for gas exchanges at Time1, Time2, and Time3, Chlorophyll Fluorescence (*Fv/Fm*; [82]) as the maximum quantum yield of photosystem II (PSII) photochemistry was registered with Handy-PEA^®^, a portable fluorometer (Hansatech Instruments, UK).

Moreover, at the same three different stages (full-veraison, maturation, and harvest), leaf predawn (ΨPD; between 03.30–04.30 a.m. on 10 fully expanded leaves per treatment) and stem midday (Ψstem; at noon o’clock on the same leaves used for leaf gas exchange measurements; leaves over 60-min dark-adapted) water potentials were estimated [83] with a model 600 pressure chamber (PMS Instrument Co., Albany, OR, USA). While in accordance to Williams and Araujo (2002) [84], leaf midday (Ψleaf) water potential was calculated as follows (r^2^ = 0.92):

Ψleaf = −0.37 + 0.91 × Ψstem(1)

Likewise, the whole-plant hydraulic conductance (Kplant) was obtained by the equation:

Kplant = E/(ΨPD − Ψleaf)(2)

As the association between the plant water depletion by transpiration (E) and the water potential dwindle from roots to leaves [85,86,87].

Finally, according to Bertamini et al., (2006) [88], 10 different leaves per treatment were used to reckon their relative water content (RWC), as follows:

RWC = ((FM − DM)/(TM − DM)) × 100(3)

where FM denotes fresh mass (leaf immediately weighed), TM denotes turgid mass (leaf reweighed placed overnight in the dark in a 25cm^3^ beaker filled with water), and DM denotes dry masses (leaf reweighed after 24 h drying at 80 °C in drying oven). The instruments used for such measurements were a dryer (Argolab TCN 30 model, MI, Italy) and a precision digital scale (FR-320 model, Gram Group Weighing Systems, Barcelona, Spain).

### 4.3. Technological Parameters of Berries

At Time1 (full-veraison; 28 July 2021), Time2 (maturation; 5 August 2021), and Time3 (harvest; 13 August 2021), 40 berries/vine sample (for repetition) was gathered from the clusters of 10 vines (10 repetitions/treatment), weighed (FR-320 model digital scale, Gram Group Weighing Systems, Barcelona, Spain), and juiced. 

With a refractometer (RF40-ND model, FLIR Extech, Munich, Germany), total sugars (°Brix) were determined. Titratable acidity (TA; gL^−1^ tartaric acid) was measured on a 10 mL sample by manual glass burette, titrating with 0.1 M NaOH to a pH 7.0 endpoint using a portable pH meter (Hanna instrument, RI, USA) [78].

### 4.4. Statistical Analysis

To compare zeolite treatment effects in disparate irrigation programs and factors interactions, all data were exposed to a two-way analysis of variance (*p* ≤ 0.05) and Tukey HSD test using R version 4.1.2. (Development for R, MA, USA). The two irrigation regimes well-watered and water stress (WWCtrl-WSCtrl) were combined with zeolite treatments and supposed as fixed factors. The data are presented as the mean ± standard deviation (sd). After running preliminary Shapiro–Wilk’s (*p* ≤ 0.05) [89] and Levene’s (*p* ≤ 0.05) [90] tests to verify the normal distribution and the homogeneity of variance of each dataset, Pearson’s linear correlation index r (*p* < 0.05) was verified to determine the strength and direction of a linear relationship between two continuous variables [91,92].

Besides, a technique for monitoring and diagnosing processes with a large and multivariate dataset, consisting of many variables with strong correlations, was elaborated (Principal component analysis, PCA) [93,94,95]. 

Graphic representations were executed by integrated development environment (IDE) RStudio software version 4.1.2. (Development for R, MA, USA) [96].

## 5. Conclusions

Maximizing fertilizer and water use efficiency in order to decrease the environmental impact of agriculture, zeolite utilization is the key to upscaling plant water holding capacity that encourages the minimizing of vine irrigation, because water is well retained within the zeolite’s structure. This experiment supplies new evidence that zeolite applications could impact both the physiological profiles and berry skin metabolism (sugar and size) of vines, giving a better skill to counteract low water availability during the season. However, it is therefore believed that further investigations should be made, above all an investigation into the activity of sugars accumulated in the vacuole (activity of sucrose-metabolizing enzymes, sucrose transporters, and monosaccharide transporters).

## Figures and Tables

**Table 1:** Two-way ANOVA (*p* < 0.05) for eco-physiology parameters in *Vitis vinifera* (Pinot Noir cv.) treated with Zeolite (t) and untreated plants (Ctrl), under two irrigation regimes (WW, well-watered; WS, water-stressed). Values are the mean of the data of each parameter, considering treatments (zeolite and Ctrl) and irrigation regime (Irrig. Regime) as factors. In the last 3 rows is indicated the significance. Other abbreviations: stomatal conductance (gs), transpiration (E), water use efficiency (WUE), fluorescence of chlorophyll (Fluo), and vapor pressure deficit (VPD). The same letter pictured on different treatments indicates no significant difference among them (mean ± SE, n = 10).

Parameter	gs	E	WUEe	Fluo	VPD
Unit	mmol m^−2^ s^−1^	mmol m^−2^ s^−1^	µmol mmol^−1^	*Fv/Fm*	kPa
**28 July 2021**					
***Treatments:***					
**WWt**	174.70 ± 55.39 a	4.62 ± 1.07 a	3.21 ± 1.16 a	0.77 ± 0.04 a	3.17 ± 0.23 b
**WSt**	145.40 ± 39.00 a	4.57 ± 1.17 a	3.00 ± 1.10 a	0.76 ± 0.05 a	3.50 ± 0.49 ab
***Irrigation regime:***					
**WWCtrl**	189.60 ± 57.25 a	5.34 ± 1.49 a	2.67 ± 0.95 ab	0.78 ± 0.03 a	3.11 ± 0.45 b
**WSCtrl**	82.60 ± 14.29 b	2.82 ± 0.29 b	1.64 ± 0.35 b	0.75 ± 0.03 a	3.76 ± 0.17 a
***Significance:***					
Treatments	0.097	0.112	0.003	1.000	0.391
Irrigation regime	0.000	0.000	0.044	0.131	0.000
Treat. × Irr. regime	0.009	0.000	0.178	0.445	0.174
**5 August 2021**					
***Treatments:***					
**WWt**	89.20 ± 22.42 a	2.28 ± 0.44 a	3.86 ± 0.53 b	0.77 ± 0.03 a	2.66 ± 0.21 c
**WSt**	69.90 ± 56.28 ab	1.85 ± 1.37 ab	4.75 ± 0.68 a	0.76 ± 0.05 a	2.81 ± 1.22 bc
***Irrigation regime:***					
**WWCtrl**	61.00 ± 18.10 b	1.61 ± 0.32 b	4.80 ± 0.87 a	0.76 ± 0.06 a	2.92 ± 0.35 b
**WSCtrl**	54.70 ± 52.66 b	1.81 ± 1.56 ab	3.63 ± 1.80 b	0.69 ± 0.04 b	3.30 ± 1.02 a
***Significance:***					
Treatments	0.000	0.007	0.639	0.010	0.000
Irrigation regime	0.038	0.376	0.422	0.007	0.000
Treat. × Irr. regime	0.281	0.015	0.000	0.090	0.098
**13 August 2021**					
***Treatments:***					
**WWt**	115.00 ± 74.64 a	3.14 ± 1.35 a	2.91 ± 1.31 a	0.77 ± 0.01 ab	4.80 ± 1.00 a
**WSt**	109.42 ± 23.12 a	2.27 ± 0.99 a	3.71 ± 0.48 a	0.76 ± 0.10 ab	3.54 ± 0.35 b
***Irrigation regime:***					
**WWCtrl**	112.30 ± 54.33 a	2.32 ± 0.84 a	3.72 ± 1.83 a	0.79 ± 0.09 a	5.04 ± 0.88 a
**WSCtrl**	47.30 ± 21.36 a	1.99 ± 0.95 a	2.26 ± 0.73 a	0.69 ± 0.10 b	3.98 ± 0.31 b
***Significance:***					
Treatments	0.078	0.187	0.498	0.347	0.209
Irrigation regime	0.056	0.150	0.484	0.024	0.000
Treat. × Irr. regime	0.105	0.522	0.020	0.035	0.711

**Table 2:** Two-way ANOVA (*p* < 0.05) for eco-physiology parameters in *Vitis vinifera* (Pinot Noir cv.) treated with Zeolite (t) and untreated plants (Ctrl), under two irrigation regimes (WW, well-watered; WS, water-stressed). Values are the mean of the data of each parameter, considering treatments (Zeolite and Ctrl) and irrigation regime (Irrig. Regime) as factors. In the last 3 rows is indicated the significance. Other abbreviations: pre-dawn water potential (ΨPD), leaf water potential (Ψleaf), stem water potential (Ψstem), and relative water content (RWC). The same letter pictured on different treatments indicates no significant difference among them (mean ± SE, n = 10).

Parameter	ΨPD	Ψleaf	Ψstem	RWC
Unit	MPa	MPa	MPa	%
**28 July 2021**				
***Treatments:***				
**WWt**	−0.27 ± 0.12 a	−1.28 ± 0.10 a	−1.00 ± 0.11 a	90.99 ± 1.76 a
**WSt**	−1.42 ± 0.22 b	−1.66 ± 0.14 b	−1.42 ± 0.16 a	88.49 ± 3.23 ab
***Irrigation regime:***				
**WWCtrl**	−0.28 ± 0.34 a	−1.28 ± 0.30 a	−1.00 ± 0.33 a	90.32 ± 3.16 ab
**WSCtrl**	−0.91 ± 0.13 b	−1.90 ± 0.11 c	−1.69 ± 0.12 b	87.32 ± 12.36 b
***Significance:***				
Treatments	0.344	0.070	0.070	0.319
Irrigation regime	0.000	0.000	0.000	0.004
Treat. × Irr. regime	0.437	0.070	0.070	0.789
**5 August 2021**				
***Treatments:***				
**WWt**	−0.51 ± 0.12 a	−1.69 ± 0.15 a	−1.45 ± 0.17 b	87.59 ± 4.70 a
**WSt**	−0.64 ± 0.22 a	−1.49 ± 0.17 a	−1.23 ± 0.19 a	82.25 ± 19.86 ab
***Irrigation regime:***				
**WWCtrl**	−0.54 ± 0.21 a	−1.57 ± 0.21 a	−1.32 ± 0.24 a	82.97 ± 5.97 ab
**WSCtrl**	−0.94 ± 0.15 b	−1.85 ± 0.15 b	−1.63 ± 0.16 c	66.70 ± 10.08 c
***Significance:***				
Treatments	0.000	0.05	0.005	0.000
Irrigation regime	0.000	0.329	0.329	0.000
Treat. × Irr. regime	0.001	0.000	0.000	0.057
**13 August 2021**				
***Treatments:***				
**WWt**	−0.36 ± 0.17 a	−1.32 ± 0.16 a	−1.05 ± 0.17 a	34.55 ± 8.22 a
**WSt**	−0.69 ± 0.13 b	−1.59 ± 0.11 b	−1.35 ± 0.12 b	28.74 ± 5.71 a
***Irrigation regime:***				
**WWCtrl**	−0.49 ± 0.21 a	−1.38 ± 0.31 a	−1.12 ± 0.34 a	29.96 ± 6.59 a
**WSCtrl**	−0.99 ± 0.18 c	−2.14 ± 0.18 c	−1.94 ± 0.20 c	26.09 ± 5.81 a
***Significance:***				
Treatments	0.000	0.000	0.000	0.144
Irrigation regime	0.000	0.000	0.000	0.069
Treat. × Irr. regime	0.135	0.000	0.000	0.662

**Table 3:** Two-way ANOVA (*p* < 0.05) for technological parameters in *Vitis vinifera* (Pinot Noir cv.) treated with Zeolite (t) and untreated plants (Ctrl), under two irrigation regimes (WW, well-watered; WS, water-stressed). Values are the mean of the data of each parameter, considering treatments (zeolite and ctrl) and irrigation regime (Irrig. Regime) as factors. In the last 3 rows is indicated the significance. The same letter pictured on different treatments indicates no significant difference among them (mean ± SE, n = 10).

Parameter	Sugar Content	Acidity	pH
Unit	°Brix	g L^−1^ tartaric ac.	pH
**28 July 2021**			
***Treatments:***			
**WWt**	15.93 ± 0.80 b	13.56 ± 1.35 a	3.12 ± 0.04 a
**WSt**	16.73 ± 0.77 a	13.13 ± 1.30 a	3.08 ± 0.10 a
***Irrigation regime:***			
**WWCtrl**	15.73 ± 0.30 b	13.56 ± 1.34 a	3.13 ± 0.04 a
**WSCtrl**	16.00 ± 0.52 ab	13.43 ± 1.09 a	3.10 ± 0.08 a
***Significance:***			
Treatments	0.024	0.736	0.466
Irrigation regime	0.010	0.526	0.053
Treat. × Irr. regime	0.187	0.736	0.916
**5 August 2021**			
***Treatments:***			
**WWt**	16.86 ± 0.67 c	12.43 ± 1.84 b	3.21 ± 0.32 a
**WSt**	17.20 ± 1.11 b	14.26 ± 3.57 a	3.13 ± 0.59 a
***Irrigation regime:***			
**WWCtrl**	16.06 ± 0.52 c	10.36 ± 0.88 c	3.17 ± 0.09 a
**WSCtrl**	17.90 ± 1.71 a	13.73 ± 0.23 a	3.00 ± 0.07 a
***Significance:***			
Treatments	0.665	0.000	0.386
Irrigation regime	0.000	0.000	0.215
Treat. × Irr. regime	0.000	0.002	0.669
**13 August 2021**			
***Treatments:***			
**WWt**	19.30 ± 0.86 a	5.36 ± 0.25 b	3.47 ± 0.05 a
**WSt**	19.98 ± 1.54 a	5.93 ± 0.48 a	3.47 ± 0.07 a
***Irrigation regime:***			
**WWCtrl**	18.22 ± 1.32 b	5.76 ± 0.35 ab	3.52 ± 0.08 a
**WSCtrl**	20.09 ± 1.42 a	5.56 ± 0.46 b	3.50 ± 0.06 a
***Significance:***			
Treatments	0.268	0.854	0.092
Irrigation regime	0.005	0.049	0.709
Treat. × Irr. regime	0.178	0.000	0.602

## Data Availability

Not applicable.
